# Boosting Drought Resilience in Rice: The Priming Effects of Zaxinone and Its Mimics

**DOI:** 10.1111/ppl.70667

**Published:** 2025-11-30

**Authors:** Teresa Mazzarella, Luca Giovannini, Guan‐Ting Erica Chen, Cristina Votta, Chiara Pagliarani, Jian You Wang, Tadao Asami, Luisa Lanfranco, Salim Al‐Babili, Raffaella Balestrini, Valentina Fiorilli

**Affiliations:** ^1^ Department of Life Sciences and Systems Biology University of Turin Turin Italy; ^2^ National Research Council Institute for Sustainable Plant Protection (CNR‐IPSP) Turin Italy; ^3^ The BioActives Lab, Center for Desert Agriculture (CDA), Biological and Environment Science and Engineering (BESE) King Abdullah University of Science and Technology Thuwal Saudi Arabia; ^4^ Graduate School of Agricultural and Life Sciences The University of Tokyo Tokyo Japan; ^5^ Center of Excellence – Sustainable Food Security King Abdullah University of Science and Technology (KAUST) Thuwal Saudi Arabia; ^6^ National Research Council Institute of Biosciences and Bioresources (CNR‐IBBR) Bari Italy

**Keywords:** abscisic acid, biostimulants, drought stress, rice, strigolactones, zaxinone, zaxinone mimics

## Abstract

Climate change increasingly threatens global agriculture, with drought emerging as a major constraint on crop productivity. Plants activate complex adaptive responses, involving genetic, physiological, and hormonal networks, to cope with water deficit. Among these, carotenoid‐derived phytohormones, abscisic acid, and strigolactones regulate drought responses through modulation of stomatal conductance, antioxidant defenses, and hormonal crosstalk. Zaxinone, an apocarotenoid metabolite, and its synthetic analogs Mimic of Zaxinone 3 (MiZax3) and MiZax5 have recently emerged as plant growth regulators and SL biosynthesis inhibitors in *Oryza sativa* . This study investigates the effects of zaxinone and its mimics on rice drought encompassing morphometric, ecophysiological, biochemical, and molecular analysis. Exogenous treatment prior to the water deficit revealed compound‐specific effects, in an organ‐specific manner, on stomatal conductance, transpiration rate, and stem water potential. We also observed differential ABA and zaxinone content and transcriptional regulation of stress‐related genes across water regimes. Notably, MiZax5 treatment maintained higher *Ψ*_stem_ and modulated stress‐responsive gene expression at the end of drought, suggesting improved osmotic adjustments and enhanced stress resilience. Our results highlight the potential of zaxinone‐based compounds as promising biostimulants capable of priming rice for improved drought resilience, offering a sustainable approach to boost crop performance under abiotic stress.

## 1 Introduction

The 2023 climate change report (IPCC 2023) underscores the extensive adverse effects of climate change, including economic losses across various sectors such as agriculture, forestry, fisheries, and energy (Calvin et al. 2023). Notably, it emphasizes the vulnerability of agricultural systems to extreme weather events, particularly drought, which represents a major threat to crop yields (Schmidhuber and Tubiello 2007; Ortiz et al. 2021). Drought stress adversely affects plant growth and physiological processes, disrupting functions at the cellular, tissue, and whole‐plant levels (Abewoy 2018; Wang, Jamil, et al. 2023; Wang, Zou, et al. 2023). To tolerate such stress, plants trigger a highly complex response governed by multiple genetic, physiological, and biochemical mechanisms (Cushman and Bohnert 2000), which encompass transcriptional, post‐transcriptional, and epigenetic changes occurring in both roots and shoot organs (Guerra et al. 2015; Forestan et al. 2020; Varotto et al. 2020).

One critical adaptation involves the regulation of root hydraulic conductivity to maintain water homeostasis (Gambetta et al. 2020; Yang et al. 2023). As soil water potential declines, a corresponding reduction in root and leaf water potential is observed, ultimately affecting the plant water balance and overall physiological performance (Liu et al. 2004). This reduction in water potential leads to tissue dehydration and loss of turgor pressure (Reddy et al. 2004), linked to a decrease in leaf area and a reduction in total aboveground biomass (Farooq et al. 2009). Drought stress also triggers excessive accumulation of reactive oxygen species (ROS), which cause oxidative damage to cells, resulting in protein denaturation and cell membrane degradation (Balestrini et al. 2019). Recent transcriptomic studies have identified a large number of drought stress‐responsive genes, including those encoding *aquaporins* (*AQPs*), *late embryogenesis abundant* (*LEA*) *proteins*, and enzymes associated with antioxidant activity, such as *superoxide dismutase* (*SOD*; Yang 2021; Wang, Chen, et al. 2024; Wang, Li, et al. 2024).

In this context, phytohormones also play a pivotal role in regulating plant responses to drought stress. Their intricate cross‐regulation initiates a cascade of signaling events that finely modulate plant growth, development, and adaptation to abiotic and biotic stimuli (Verma et al. 2016; Yang 2021; El Sabagh et al. 2022). Among these hormones, abscisic acid (ABA), a carotenoid‐derived phytohormone synthesized by 9‐cis‐epoxycarotenoid dioxygenase (NCED) enzymes, acts as a central signaling molecule that regulates drought stress responses, primarily due to its role in stomatal closure, which reduces stomatal conductance and minimizes transpiration losses (De Ollas and Dodd 2016; Balestrini et al. 2018; Rai et al. 2024). Additionally, ABA contributes to drought tolerance by promoting cuticle thickening, further limiting water loss (Bi et al. 2017). However, increasing evidence suggests that drought stress responses in various plant species involve a more complex and coordinated phytohormonal signaling network (Salvi et al. 2021). Indeed, strigolactones (SLs), another class of carotenoid‐derived phytohormones synthesized through the enzymatic activity of Dwarf 27 (D27), Carotenoid Cleavage Dioxygenase 7 (CCD7), and CCD8, play a crucial role in regulating plant architecture, development, interactions with rhizosphere microbial community, and contribute to abiotic stress adaptation by enhancing antioxidant responses (Cardinale et al. 2018; Felemban et al. 2019; Wu et al. 2022, Wang, Chen, et al. 2024; Wang, Li, et al. 2024). SL‐depleted plants are hypersensitive to drought due to stomatal hyposensitivity to ABA, suggesting that SLs contribute to drought adaptation (Liu et al. 2015; Haider et al. 2018). However, evidence showed that their signaling can be either ABA‐dependent or independent (Cardinale et al. 2018; Trasoletti et al. 2022). The beneficial role of SLs in drought tolerance has also been demonstrated through the exogenous application of GR24, a SL synthetic analog, which alleviates drought stress in various plant species by improving water relations, increasing osmolyte accumulation, maintaining chlorophyll content, and boosting both enzymatic and non‐enzymatic antioxidant activities (Ha et al. 2014; Min et al. 2019; Visentin et al. 2020; Sattar et al. 2022; Song et al. 2023).

Another carotenoid‐derived molecule, zaxinone, has been shown to promote plant growth by acting as a negative regulator of SL biosynthesis and influencing the recruitment of root microbiota communities in rice (Wang et al. 2019, 2021; Votta et al. 2022; Mazzarella et al. 2024). Its growth‐promoting effects are linked to enhanced root sugar uptake and metabolism, and to the modulation of cytokinin homeostasis (Wang et al. 2021). Due to the complex synthesis of zaxinone, easily synthesized mimics, namely Mimic of Zaxinone 3 (MiZax3) and 5 (MiZax5), have been developed. These mimics show comparable biological activities, including the promotion of rice growth, reduction of SL levels, and mitigation of *Striga* infestation (Wang et al. 2019, 2021). Exogenous application of zaxinone and MiZax led to benefits in several horticultural crops under both normal and desert field conditions (Wang et al. 2020, 2022). Nonetheless, more experimental data are needed to determine and understand the biological effects of these molecules in plants, especially in response to abiotic stress. To foster the application of these novel growth‐promoting regulators as biostimulants under field conditions, we investigated their role in rice responses to water deficit. For this purpose, we explored their potential in priming rice against drought stress individually by treating plants with zaxinone, MiZax3, or MiZax5 before imposing water stress. To gain an in‐depth picture of rice responses to the imposed treatments, we employed a multidisciplinary approach, which includes morphometric, ecophysiological, biochemical, and molecular analysis. Our findings demonstrate that the application of zaxinone and MiZax showed different effects on ecophysiological parameters, the expression of stress‐responsive genes, and the content of hormones associated with water stress, in a tissue‐specific manner and according to water availability. Among the tested compounds, MiZax5 consistently produced the most pronounced beneficial effects, enhancing drought tolerance by increasing stem water potential and modulating the expression of stress‐responsive genes.

## 2 Materials and Methods

### 2.1 Plant Material and Growth Conditions

Rice seeds ( *Oryza sativa* subsp. *Japonica* cv Nipponbare) were surface sterilized in 50% (v/v) household bleach for 15 min, rinsed five times with distilled water, and germinated in plastic pots (*V* = 1530 cm^3^ = 1.53 L). The pots were filled with 1:1 (v/v) of the 0.8 mm sterilized quartz sand and native paddy soil harvested from fields of the CREA‐CI Research Centre (Vercelli, Italy), whose physico‐chemical properties and microbial community were already characterized in previous studies (Chialva et al. 2020; Hester et al. 2022; Mazzarella et al. 2024). Plants were grown in a greenhouse facility from 25th July 2023 to 25th September 2023 (60 days). During the experimental period, the daily temperature and relative humidity conditions averaged around 24.9°C ± 5.35°C and 42.3%–61.8%, respectively, while the maximal photosynthetic flux density (PPFD) within the greenhouse was 850–1100 μmol photons m^−2^ s^−1^. After 10 days from sowing, plants were treated individually with different compounds by pouring into the substrate 50 mL per pot of tap water containing 5 μM (10^−6^) zaxinone (Zax), MiZax3, or MiZax5 dissolved in 0.1% acetone, and the corresponding volume of the acetone (ACE) solvent (control), once a week for a total of six applications. We used two‐month‐old plants (80/90 cm shoot height), which were still in the vegetative stage before the stress imposition. Out of the 30 plants for each compound treatment, we used 10 as a control (not stressed and maintained in a well‐watered condition, WW). The remaining 20 plants were subjected to water stress (WS) treatment by withholding irrigation for a total of 6 days until reaching a severe stress condition. Then, for a subset of 10 plants, a 7‐day rehydration period followed, allowing the plants to recover (R).

### 2.2 Biometric and Eco‐Physiological Parameters

**FIGURE 1:**
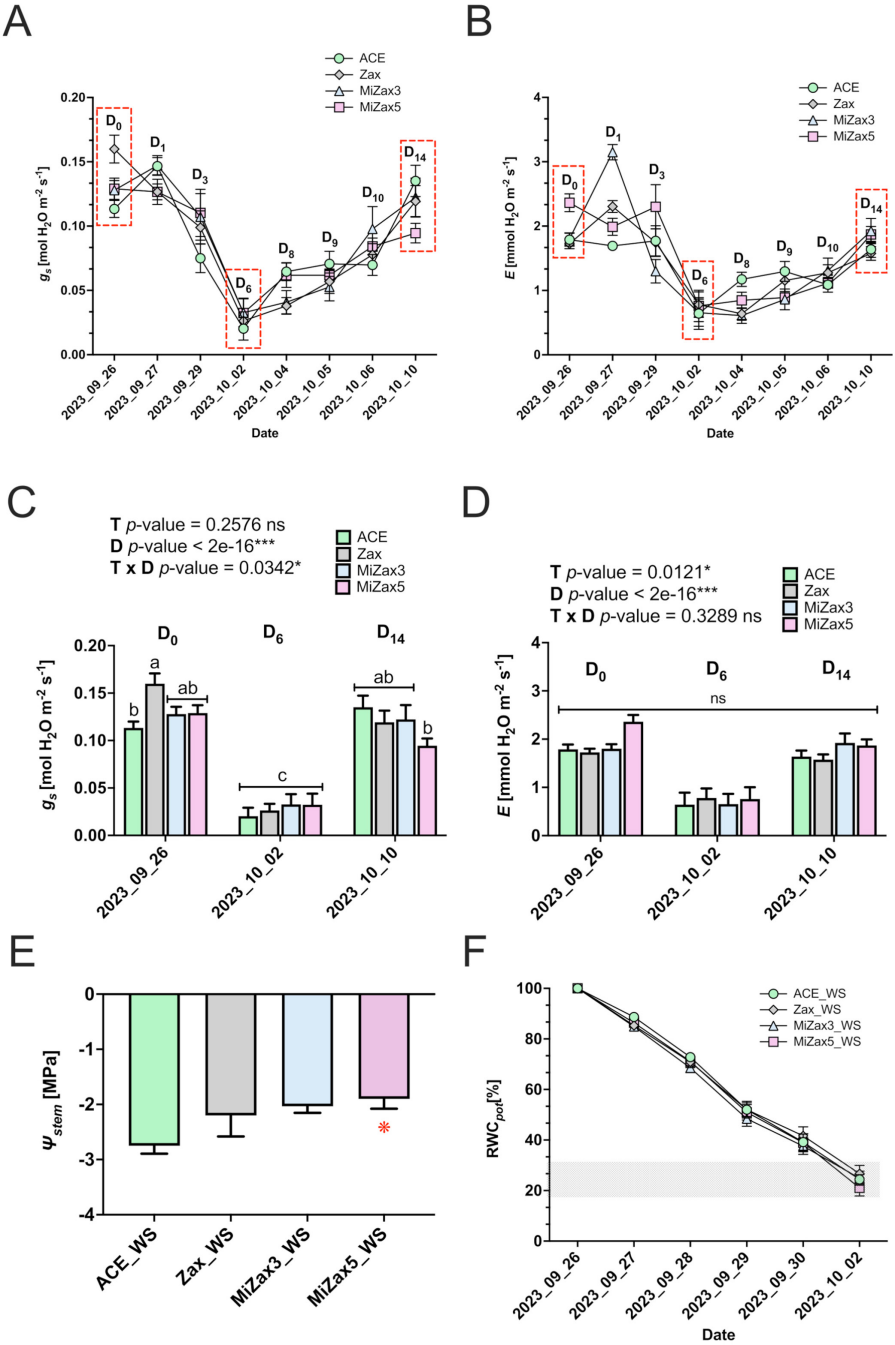
Effects of treatment with Zax, MiZax3, and MiZax5 on the physiological responses of potted rice plants during a water stress (WS) and recovery (R) time‐course. (A) Dynamic changes in stomatal conductance (*g*_*s*_, mol H_2_O m^−2^ s^−1^) and (B) leaf transpiration (*E*, mmol H_2_O m^−2^ s^−1^) monitored throughout the entire duration of the water stress period and subsequent recovery. Red dashed rectangles indicate the beginning of the stress (D_0_), the end of the stress (D_6_), and the end of recovery (D_14_). (C, D) *g*_*s*_ and *E* rates measured at the beginning (D_0_), at the end of the stress period (D_6_), and on the final day of recovery (D_14_). (E) Stem water potential (*Ψ*_stem_) measured in treated plants at the end (D_6_) of the WS. (F) Changes in soil relative water content (RWC_soil_, %) monitored throughout the water stress period (WS). All results are presented as mean ± SE. Statistically significant differences were determined using a two‐way ANOVA test, considering the interaction between treatment (T) and days (D). Different lowercase letters indicate significant differences among groups and are reported only when the T × D interaction is significant, as assessed by the Tukey's HSD test (*p* < 0.05). ns, not significant. A red asterisk denotes cases where a specific treatment significantly differed from the control (ACE), as determined by a pairwise comparison *t*‐test (*p < 0.05), as follows: *p ≤ 0.05, **p ≤ 0.01, ***p ≤ 0.001. Analysis of variance on the single variables is reported in Tables S2–S5. ACE, control; MiZax3 and MiZax5, synthetic zaxinone mimics; R, recovery; WS, water stress; WW, well‐watered; Zax, zaxinone treatment.

The water deficit period began on September 26, 2023 (D_0_) and ended on October 02, 2023 (D_3_). On the first and last experimental day, we recorded the plant height and tillering. We monitored stomatal conductance (*g*_*s*_) and transpiration rates (*E*) every morning using adult, non‐senescing leaves at the same physiological age. Measurements were taken in randomized rotation using a portable porometer/fluorometer (Ll‐600, LI‐COR) between 9:00 and 12:00 a.m. to maintain the same light conditions of the leaf. Daily changes in water loss were also inspected by measuring the soil relative water content (RWC_soil_, %) gravimetrically on potted plants exposed to water deprivation according to Pagliarani et al. (2022). The water stress lasted 6 days and ended when RWC reached around 25% of its initial value and *g*_*s*_ values were equal to or lower than 0.05 mol H_2_O m^−2^ s^−1^. Once water stress was reached, part of the WS and WW plants (10 biological replicates per thesis per condition), roots, and leaves were sampled for subsequent analysis. Subsequently, unsampled plants (10 biological replicates per condition) were rehydrated and monitored physiologically until the complete recovery of gas exchange parameters (i.e., October 10, 2023; D_7_; Figure 1A). The Chlorophyll Content Index (CCI) was measured on three leaves for each plant and considering all plants for each thesis. The measurement was repeated three times during the whole trial duration, September 26, 2023 (D_0_), October 2, 2023 (D_3_), and October 10, 2023 (D_8_), using a portable chlorophyll meter (SPAD 502; CCM‐200; Opti‐Sciences). Additionally, the stem water potential (*Ψ*_stem_) was measured on WS plants at the end of the trial (D_3_), as described in Pagliarani et al. (2022), using a portable pressure chamber (1505D PMS Instrument Company). The analysis of *Ψ*_stem_ was carried out on four biological replicates for the WS group.

### 2.3 Metabolite Extraction and Quantification

Zaxinone was extracted according to Wang et al. (2019), and D3‐zaxinone (customized synthesis; Buchem B.V., Apeldoorn, the Netherlands) was used as an internal standard. Quantification of endogenous hormones was performed following the procedure of Chen et al. (2023). Briefly, 15 mg freeze‐dried ground root or shoot tissues were spiked with internal standards D3‐zaxinone (10 ng) and D6‐ABA (10 ng) along with 750 μL of methanol. The mixture was sonicated for 15 min in an ultrasonic bath (Branson 3510 ultrasonic bath), followed by centrifugation for 5 min at 14000 × g at 4°C. The supernatant was collected, and the pellet was re‐extracted with 750 μL of the same solvent. Then, the two supernatants were combined and dried under vacuum. The sample was redissolved in 100 μL of acetonitrile: water (25:75, v‐v) and filtered through a 0.22‐μm filter for LC–MS analysis. Plant hormones were analyzed by LC–MS/MS using the UHPLC‐Triple‐Stage Quadrupole Mass Spectrometer (Thermo ScientificTM Altis). Chromatographic separation was achieved on the Hypersil GOLD C18 Selectivity HPLC Columns (150 × 4.6 mm; 3 μm; Thermo ScientificTM) with mobile phases consisting of water (A) and acetonitrile (B), both containing 0.1% formic acid and the following linear gradient (flow rate, 0.5 mL min^−1^): 0–10 min, 15%–100% B, followed by washing with 100% B for 5 min and equilibration with 15% B for 2 min. The injection volume was 10 μL, and the column temperature was maintained at 35°C for each run. The MS parameters of Thermo Scientific Altis were as follows: positive mode for zaxinone and negative mode for ABA, ion source of H‐ESI, ion spray voltage of 3000 V, sheath gas of 40 arbitrary units, aux gas of 15 arbitrary units, sweep gas of 0 arbitrary units, ion transfer tube gas temperature of 350°C, vaporizer temperature of 350°C, collision energy of 20 eV, CID gas of 2 mTorr, and full width at half maximum (FWHM) 0.4 Da of Q1/Q3 mass. The characteristic Multiple Reaction Monitoring (MRM) transitions (precursor ion → product ion) were 275.2 → 239, 275.2 → 257 for zaxinone; 278.1 → 242, 278.1 → 260 for D3‐zaxinone; 176.2 → 219.1 for ABA; 269.2 → 159.1 for D6‐ABA.

### 2.4 Quantitative Gene Expression Analysis of Leaves and Roots

Expression changes of the target transcripts were quantified in leaf and root samples (five independent biological replicates) by RT‐qPCR. Leaves and roots were immediately frozen in liquid nitrogen and stored at –80°C. Total RNA was extracted from each biological replicate, starting from 100 mg of material and using the Plant RNeasy Kit (Qiagen), according to the manufacturer's instructions. RNA quantity was checked using a NanoDrop 1000 spectrophotometer (Thermo Fisher Scientific). Samples were treated with TURBO DNase (Thermo Fisher Scientific) according to the manufacturer's instructions. The RNA samples were routinely checked for DNA contamination by PCR analysis, using *OsRubQ1*. For single‐strand cDNA synthesis, total RNA was denatured at 65°C for 5 min and then reverse‐transcribed at 25°C for 10 min, 42°C for 50 min, and 70°C for 15 min. The final volume was 20 μL and contained 10 μM random primers, 0.5 mM dNTPs, 4 μL 5X buffer, 2 μL 0.1 M DTT, and 1 μL Superscript II (Invitrogen). Quantitative RT‐PCR (RT‐qPCR) was performed using the Connect Real‐Time PCR Detection System (Bio‐Rad Laboratories). Each PCR reaction was carried out in a total volume of 10 μL, containing 1 μL of diluted cDNA (1:3), 5 μL of 2X SYBR Green Reaction Mix, and 0.2 μL of each primer (10 μM). The following PCR program was used: an initial denaturation phase at 95°C for 3 min, 40 cycles at 95°C for 10 s, and annealing at gene‐specific temperatures for 30 s. A melting curve at 0.5°C for 5 s was recorded at the end of each run to exclude the generation of non‐specific PCR products (Ririe et al. 1997). All reactions were performed on at least five biological and two technical replicates. The expression of rice target transcripts was quantified after normalization to two reference genes: *OSRubQ1* encoding a ubiquitin‐protein and *OsAct1* encoding an actin protein (Lee et al. 2010; primers are listed in Table S1).

### 2.5 Statistical Analyses

Statistical analysis was performed using R software (version 4.1.1). To identify the differences among treatments (T), stress conditions (S), or days of stress (D) and their interactions (T × S or S × D), a two‐way ANOVA test was applied. A two‐way ANOVA was also applied to assess the effect of treatment on physiological performance (*g*_*s*_, *E*) during the period of WS and subsequent rehydration (R). The standard error (SE) of the mean was calculated for all data. Significant differences between means were checked by Tukey's HSD test (*p* ≤ 0.05) and are reported in the figure panels only when the interaction T × S (or D × S) was significant. Furthermore, to assess the differences between the treatments and the control (ACE), the pairwise comparison *t*‐test was applied within each stress condition (WW, WS, R), comparing the individual treatments with their respective controls. Graphs were generated using the Prism v.9.5.0 software (La Jolla, CA, USA v.9.5.0).

## 3 Results

### 3.1 Effect of Exogenous Application of Zaxinone and MiZax on Rice Eco‐Physiological Responses Upon Water Deficiency

To determine the effect of the compounds, we treated all plants once per week with ACE (acetone control), Zax, MiZax3, or MiZax5 for 6 weeks. Fifty days after germination, plants were exposed to either WW or WS conditions, while the R was initiated on half of the WS plants following 6 days of water withdrawal. At the beginning of the stress imposition (D_0_), statistically significant differences among treatments were evident, with Zax presenting the highest stomatal conductance (*g*_*s*_) values and MiZax5 the highest transpiration (*E*) rates (Figure 1A,B; Table S2), compared to the other plant groups.

During the whole water deprivation period, WS plants encountered a progressive reduction in *g*_*s*_ and *E* levels, following a similar trend among the treatments (Figure 1A,B; Table S2). The same response was also observed during the rehydration phase, in which *g*_*s*_ and *E* rates gradually increased until recovery completion (Figure 1A,B; Figure S1).

It is worth noting that treatment (T) had no significant effect on *g*_*s*_, which, conversely, was significantly influenced by time (D) and the T × D interaction (Figure 1C; Table S3). In contrast, *E* was only significantly affected by T and D (Figure 1D; Table S3). Notably, after 3 days of water deprivation (D_3_), plants treated with Zax, MiZax3, and MiZax5 showed a trend towards higher *g*_*s*_ values (1.4 times) compared to the control plants (ACE; Figure 1A; Table S2). These differences decreased at the end of the stress period (D_6_; October 2nd, 2023), although *g*_*s*_ values in the treated plants were slightly higher compared to the ACE controls (0.3 mol H_2_O m^−2^ s^−1^ vs. 0.2 mol H_2_O m^−2^ s^−1^; Figure 1A; Table S2). Regardless of the treatment, plants on the last day of stress (D_6_; October 2nd, 2023) showed a statistically significant reduction in *g*_*s*_ and *E* compared to the first day of stress (D_0_; September 26th, 2023; Figure 1C,D; Table S3).

This result was also confirmed by comparing *g*_*s*_ and *E* rates measured in WS plants to those recorded in WW controls at the end of the WS imposition (Figure S1A,B; Table S4). Moreover, at the end of the stress (D_6_; October 2, 2023), the plants showed a stem water potential (*Ψ*_stem_) that tended to be more negative than in WW plants (Figure 1E; Table S5), confirming the effect of the stress treatment.

Notably, plants treated with MiZax5 showed a significantly higher *Ψ*_stem_ (averaging around −1.9 MPa) than the other treated plants, including the control ones (ACE). Zax and MiZax3‐treated plants, although not statistically significant, also showed a trend towards higher *Ψ*_stem_ than ACE, with values close to −2.2 MPa (Figure 1E; Table S5). These data were in agreement with what emerged from the trend of leaf gas exchanges during the last day of water shortage (D_6_; October 2nd, 2023), particularly for the MiZax5 and MiZax3 treatments, which displayed higher, though not statistically significant, *g*_*s*_ rates than ACE‐treated plants. Furthermore, no significant changes were observed by inspecting the soil water loss dynamics with the calculation of RWCpot (%). In fact, independently of the treatment, all plants reached RWC pot values of about 20%–30% (Figure 1F). At the end of the imposed stress period, half of the plants were rehydrated, still showing remarkable differences in eco‐physiological parameters compared to WW plants after 2 days of rehydration (D_8_; 4 October 2023; Figure S1; Table S2). After the rehydration period (D_14_; October 10th, 2023), all plants were successful in restoring their physiological function, with *g*_*s*_ and *E* rates being significantly higher than those measured on the last day of stress (D_6_; October 2nd, 2023; Figure 1A–D; Table S3), showing stomatal conductance values comparable to those of WW plants (Figure S1; Table S2). In detail, all plants stabilized at *g*_*s*_ values around 0.10–0.15 mol H_2_O m^−2^ s^−1^ (D_14_; October 10, 2023; Figure 1A–D), comparable to those of WW plants (Figure S1; Table S4). After 7 days of rehydration, representing the last day of recovery (D_14_; October 10, 2023), MiZax5‐treated plants showed *g*_*s*_ rates lower than those recorded on the other plant groups; however, such differences were not statistically significant (Figure 1A; Table S2). Biomass analysis revealed that treatment with Zax and MiZax3 led to an increase in the dry biomass shoot compared to ACE in WW conditions. Conversely, no differences in this parameter were observed upon WS as well as in terms of root dry biomass, regardless of the applied water regime (Figure S2 and Table S6). Unlike the shoot dry weight, the CCI was overall affected by the timing of stress imposition (D) but not by the treatments (T). However, some differences ascribed to specific treatments emerged at D_0_ (September 26th, 2023), as leaves of Zax‐treated plants showed a significantly lower CCI compared to ACE control plants. Furthermore, after 7 days of rehydration (D_14_; October 10th, 2023) CCI values were significantly higher in MiZax3‐treated plants with respect to ACE (Figure S2C and Table S7). Taken together, these data suggest that plant treatments with Zax, MiZax3, and MiZax5 exert a mid‐term priming effect, and that MiZax5 may improve the osmotic adjustment capacity compared to non‐treated plants.

### 3.2 Tissue‐Specific Variation in Abscisic Acid and Zaxinone Content

To assess the effects of the exogenous treatments on the drought‐related hormone ABA and the endogenous ZAX content, we quantified the levels of both rice leaves and roots subjected to WW, WS, and R.

**FIGURE 2:**
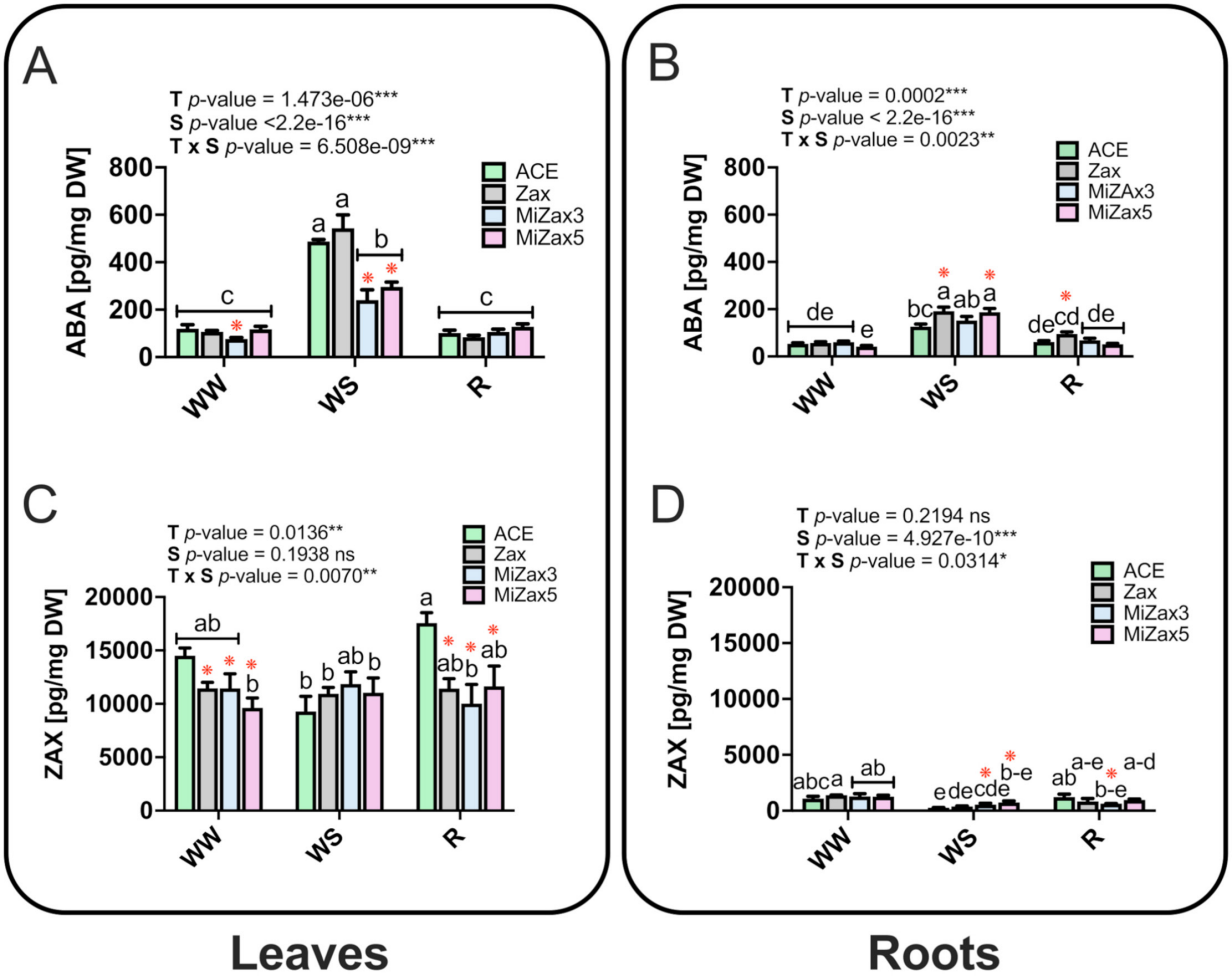
Metabolite content in rice leaves and roots. The plants were sampled on the last day of stress, under well‐watered (WW) and water‐stressed (WS) conditions, and on the last day of recovery (R). (A, B) ABA concentrations in leaves and roots, and (C, D) Zax concentrations in leaves and roots, respectively. Treatments are reported in greyscale according to the color legend to the right of the graph (ACE, acetone control; Zax, zaxinone; MiZax3 and MiZax5, synthetic zaxinone mimics). Data are expressed as pg mg^−1^ DW. All results are reported as mean ± SE. Statistically significant differences were determined using a two‐way ANOVA test, considering the interaction between treatment (T) and stress condition (S). Lowercase letters above the bars denote significant differences, assessed by the Tukey's HSD test (*p* < 0.05), and are reported only when the T × S interaction is significant. ns, not significant. Red asterisks indicate significant differences compared to control plants (ACE) within each stress condition (WW, WS, R), as assessed by the pairwise comparison *t*‐test (*p* < 0.05), as follows: *p ≤ 0.05, **p ≤ 0.01, ***p ≤ 0.00. Analysis of variance on the single variables is reported in Table S8.

ABA levels were significantly influenced by both T and S as well as by their interaction (T × S; Figure 2A,B; Table S8). In detail, a significant increase was observed under WS conditions compared to WW samples in both roots and leaves. Following WS, ABA accumulation was much less accentuated in the leaves of MiZax3 and MiZax5‐treated plants than in ACE and Zax‐treated plants (Figure 2A). In roots, both Zax and MiZax5 induced an increase in ABA content upon WS conditions (Figure 2B). At the end of the recovery period, ABA content measured in leaves and roots returned to pre‐stress values in all treatments (Figure 2A), although significantly higher concentrations were quantified in the roots of Zax‐treated plants with respect to ACE (Figure 2B). Notably, the analysis of ZAX content revealed tissue‐specific accumulation patterns with concentration levels varying according to the treatment, mainly in WW and R leaves and in WS and R roots (Figure 2C,D; Table S8). Under WW conditions, ZAX content significantly decreased in the leaves of all treated plants, compared to those of ACE. The same result was also evident during recovery, whereas no notable differences were detected among the diverse treatments under WS. These accumulation patterns were partially reversed in the roots. Indeed, WS significantly affected the root ZAX content, leading to a significantly lower amount than those measured in WW conditions, with the only exception of MiZax5‐treated samples (Figure 2D). Some treatment‐specific changes were also observed in R conditions. Particularly, MiZax3 and MiZax5‐treated plants showed significantly higher ZAX content under WS conditions, while treatment with MiZax3 significantly reduced the metabolite content during recovery. Collectively, these results suggest that the exogenous application of the selected compounds influences both ABA and ZAX accumulation profiles to different extents based on the plant organ and water regime conditions.

### 3.3 Expression of Stress‐Responsive Genes Under Water Stress Upon Zax and MiZax Treatments

**FIGURE 3:**
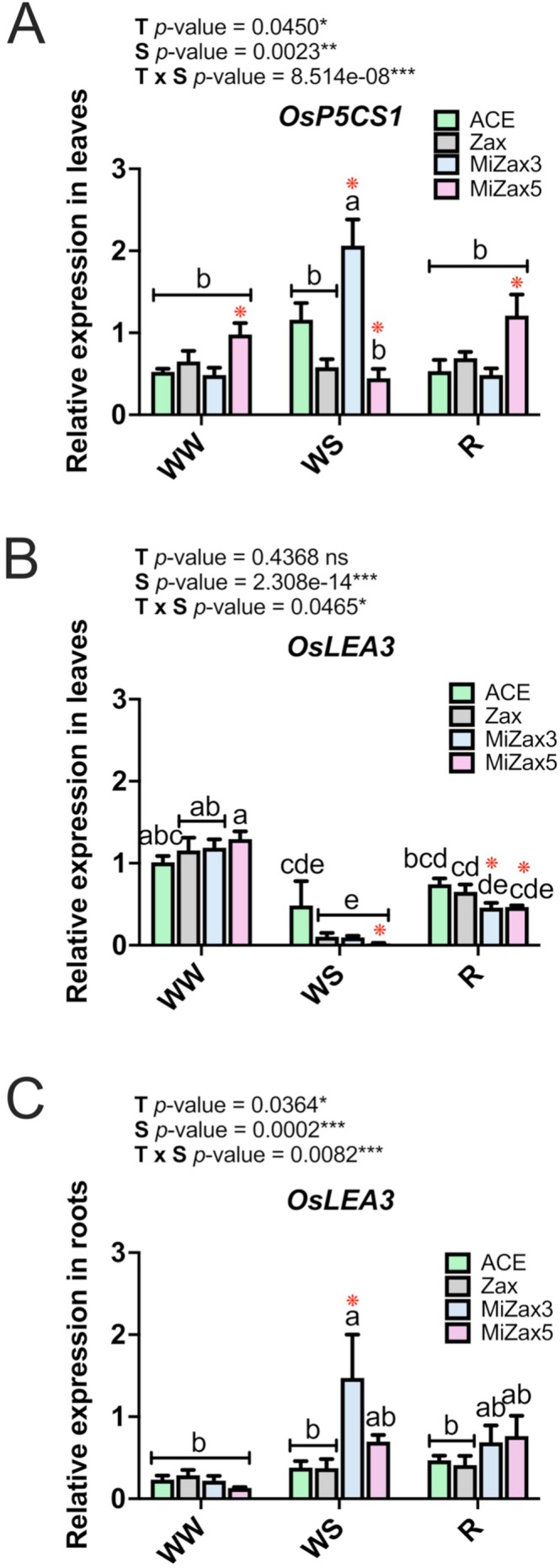
Relative expression levels of stress‐responsive genes in rice leaves and roots. *OsP5CS1* and *OsLEA3* in leaves, and *OsLEA3* in roots, under well‐watered (WW), water‐stressed (WS), and recovery (R) conditions. Treatments are reported in greyscale according to the color legend to the right of the graph (ACE, acetone control; Zax, zaxinone; MiZax3 and MiZax5, synthetic zaxinone mimics). All results are reported as mean ± SE. Statistically significant differences were determined using a two‐way ANOVA test, considering the interaction between treatment (T) and stress condition (S). Lowercase letters above the bars denote significant differences, assessed by the Tukey's HSD test (*p* < 0.05), and are reported only when the T × S interaction is significant. ns, not significant. Red asterisks indicate significant differences compared to control plants (ACE) within each stress condition (WW, WS, R), as assessed by the pairwise comparison *t*‐test (*p* < 0.05), as follows: *p ≤ 0.05, **p ≤ 0.01, ***p ≤ 0.00. Analysis of variance on the single variables is reported in Tables S9 and S10.

To elucidate the organ‐specific effects of exogenous treatments on the expression of drought‐marker genes, we examined transcript levels of stress‐inducible genes in rice leaves (Figure 3A,B; Figure S3A,B; Table S9) and roots (Figure 3C; Figure S3C,D; Table S10) across the different water availability treatments. Particularly, the expression of *OsP5CS1* (*LOC_Os05g38150*), which encodes for a key enzyme involved in proline biosynthesis, transcriptionally induced by salt, dehydration, and cold stress (Loscos et al. 2006; Szabados and Savouré 2010; Forlani et al. 2015), and *OsLEA3* (*LOC_Os05g46480*), which encodes a Late Embryogenesis Abundant protein involved in cellular protection during desiccation stress (Xiao et al. 2007; Duan and Cai 2012) were evaluated. In WW leaves, *OsP5CS1* expression significantly increased only in MiZax5‐treated plants in comparison with ACE controls (Figure 3A). In WS conditions, an opposite expression pattern was observed in MiZax5‐treated plants, which showed a significant decrease in *OsP5CS1* transcript abundance compared to ACE‐treated plants. Conversely, the highest *OsP5CS1* expression rates were detected in the leaves of WS MiZax3‐treated plants. At the end of the recovery phase, *OsP5CS1* expression profiles were similar to those observed in treated plants under WW conditions, with MiZax5 showing significantly higher transcription rates compared to ACE_R (Figure 3A). On the other hand, regardless of the specific treatment, *OsLEA3* transcript levels in leaves were significantly reduced under both WS and R compared to WW conditions (Figure 3B). Additionally, after WS imposition, the *OsLEA3* gene was strongly down‐regulated in MiZax5‐treated plants compared to the ACE‐treated ones, whereas during recovery, both MiZax treatments reduced the expression of this gene in leaves (Figure 3B). In the roots, *OsLEA3* was upregulated following MiZax3 treatment (Figure 3C) and in association with WS, while no significant differences among treatments were recorded during WW and R conditions. Overall, these data showed that Zax and MiZax treatments modulated drought gene expression differently in leaves and in roots. In particular, MiZAX5 seems to precondition plant response to drought stress in WW by inducing *OsP5CS1* in leaves, while MiZAX3 exerted a more pronounced impact on the *OsP5CS1* and *OsLEA3* transcripts under WS in both organs.

### 3.4 Expression Patterns of ABA and Ethylene‐Related Genes in Rice Leaves and Roots

**FIGURE 4:**
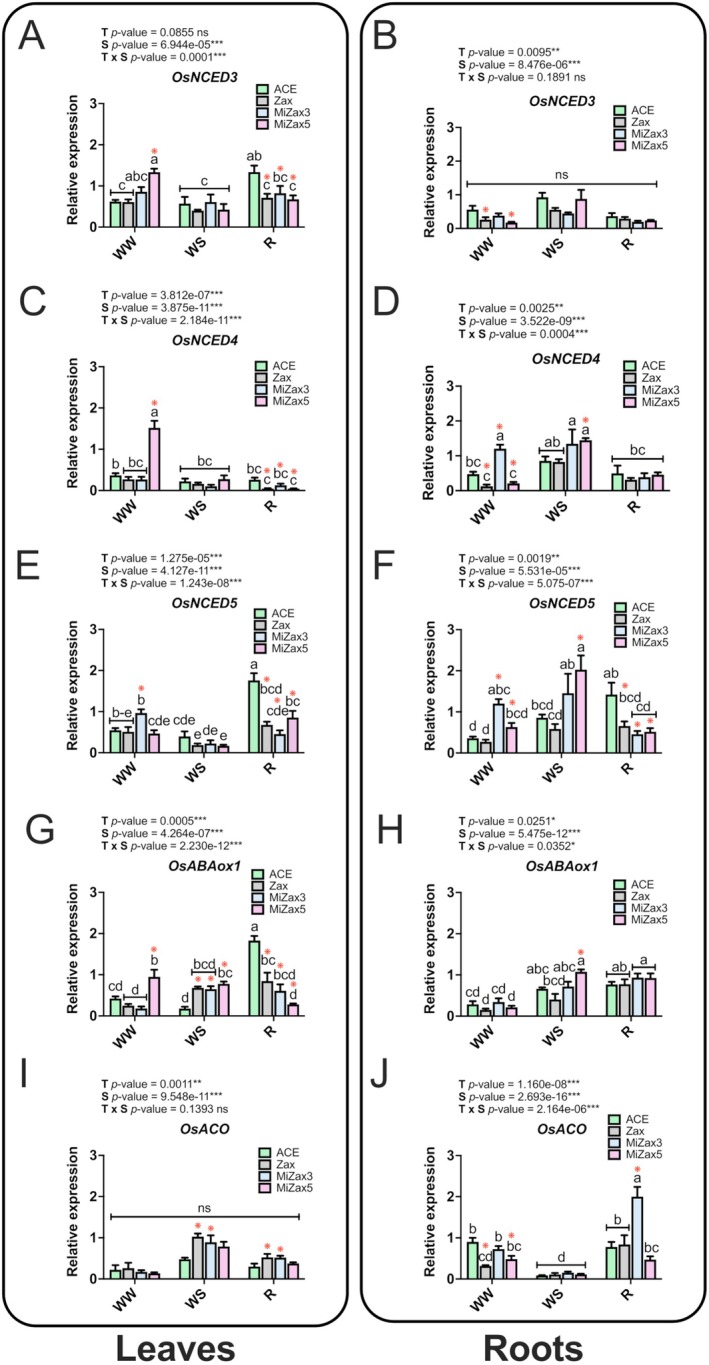
Relative expression of stress hormone‐related genes in rice leaves and roots. Genes involved in ABA biosynthesis and degradation (*OsNCED3*, *OsNCED4*, *OsNCED5*, *OsABAox1*) and ethylene biosynthesis (*OsACO*) were investigated in leaves and roots of rice plants under well‐watered (WW), water‐stressed (WS), and recovery (R) conditions. Treatments are reported in greyscale according to the color legend to the right of the graph (ACE, acetone control; Zax, zaxinone; MiZax3 and MiZax5, synthetic zaxinone mimics). All results are reported as mean ± SE. Statistically significant differences were determined using a two‐way ANOVA test, considering the interaction between treatment (T) and stress condition (S). Lowercase letters above the bars denote significant differences, assessed by the Tukey's HSD test (*p* < 0.05), and are reported only when the T × S interaction is significant. ns, not significant. Red asterisks indicate significant differences compared to control plants (ACE) within each stress condition (WW, WS, R), as assessed by the pairwise comparison *t*‐test (*p* < 0.05), as follows: *p ≤ 0.05, **p ≤ 0.01, ***p ≤ 0.00. Analysis of variance on the single variables is reported in Tables S9 and S10.

To evaluate the impact of the treatments on hormone‐related gene expression, we quantified the relative expression of six genes involved in ABA biosynthesis and catabolism and in ethylene (ET) biosynthesis. Expression was assessed in rice leaves and roots collected from control (ACE) and treated plants (Figure 4A–J; Tables S9 and S10), as these genes are differentially regulated in these organs (Sharp 2002). Particularly, the *NCED* gene family catalyzes key steps in ABA biosynthesis (Seo and Koshiba 2002). Here, the expression levels of *OsNCED3*, *OsNCED4*, and *OsNCED5* were evaluated. *OsNCED3 (LOC_Os03g44380)*, which was reported to be constitutively expressed in various tissues of rice and involved in abiotic stress tolerance (Huang et al. 2018), was not differentially expressed in WS leaves compared to WW (Figure 4A). However, WW MiZax5‐treated leaves showed a significant up‐regulation of this gene with respect to ACE and Zax, as well as to leaves of plants treated with the same compound but subjected to WS. Additionally, during recovery, *OsNCED3* was down‐regulated in the leaves of all treated plants compared to ACE (Figure 4A). In roots, *OsNCED3* expression was not differently modulated among the treatments, independently of the water availability, except for WW Zax‐ and WW MiZax5‐treated samples, which showed a significant downregulation of the gene compared to WW ACE controls (Figure 4B). *OsNCED4* (*LOC_Os07g05940*), which was already reported to confer multi‐abiotic stress tolerance in rice (Xiang et al. 2024), was strongly induced under WW upon MiZax5 and MiZax3 treatment in leaves and roots, respectively (Figure 4C,D). In leaves, *OsNCED4* expression was not differentially affected by water regime (WW, WS, and R) in ACE‐treated samples, although treatment with Zax and its mimics led to a significant downregulation at the end of the recovery in comparison to ACE (Figure 4C).

*OsNCED4* expression in roots (Figure 4D) displayed a significant increase under WS compared to WW conditions only upon Zax and MiZax5 treatments. It is worth noting that in WW plants, the treatment with MiZax3 induced a significantly higher expression of *OsNCED4* compared to Zax and MiZax5. *OsNCED5* (*LOC_Os12g42280*), recognized as a gene associated with enhanced tolerance to drought, salt, and osmotic stress (Huang et al. 2019), was upregulated in leaves of WW MiZax3‐treated plants compared to ACE (Figure 4E). Additionally, despite the interaction between water deprivation and any of the imposed treatments not affecting *OsNCED5* transcription, a significant down‐regulation of this gene occurred during recovery in all treated plants compared to ACE. In roots, both MiZax3 and MiZax5 increased the expression of *OsNCED5* in comparison to ACE and Zax, especially during WW and WS conditions (Figure 4F). It is relevant that in R plants, all the Zax/MiZax treatments inhibited the expression of this gene with respect to ACE plants (Figure 4F). *OsABAox1* (*LOC_Os02g47470*) encodes for an ABA 8′‐hydroxylase, that is, an enzyme responsible for ABA degradation, which contributes to ABA homeostasis regulation (Liu et al. 2022). In leaves, only treatment with MiZax5 enhanced the expression of this gene upon irrigation, while during WS, all treatments significantly induced *OsABAox1* transcription. Notably, an opposite trend was observed in R conditions (Figure 4G), highlighting the impact of the T × S interaction in the modulation of such responses. In roots, *OsABAox1* expression increased switching from WW to WS conditions, although changes were significant only in MiZax5‐treated plants (Figure 4H). Though no differences ascribed to the specific treatment were noticed upon R, *OsABAox1* was still upregulated in root samples collected at the end of recovery with respect to WW plants, suggesting a role in the maintenance of ABA homeostasis under both stress and recovery conditions. *OsACO* (*LOC_Os01g39860*), encoding 1‐aminocyclopropane‐1‐carboxylate oxidase, a key enzyme catalyzing the final step of the ethylene biosynthetic pathway (Khan et al. 2024), was also analyzed. The transcript of *OsACO* was upregulated in leaves under WS and R conditions but exclusively in the presence of Zax and MiZax3 (Figure 4I). In roots, *OsACO* expression was significantly reduced in WW Zax and MiZax5‐treated plants (Figure 4J). Conversely, during WS imposition, its expression decreased across all treatments, including the ACE control. Although the *OsACO* expression levels increased during recovery, approaching those of WW conditions, only the MiZax3 treatment positively affected gene transcription (Figure 4J), further attesting to the importance of the T × S interaction in regulating such molecular signals. Our results indicated that all treatments modulated ABA biosynthesis and catabolism in both leaves and roots under different water regimes, while negatively impacting ethylene biosynthesis in roots in WW conditions. Moreover, the data suggested that MiZax5 preconditioned the leaves of WW plants by upregulating the expression of genes associated with ABA synthesis and catabolism.

### 3.5 Role of SL and Zaxinone Biosynthesis in Drought Stress Response

**FIGURE 5:**
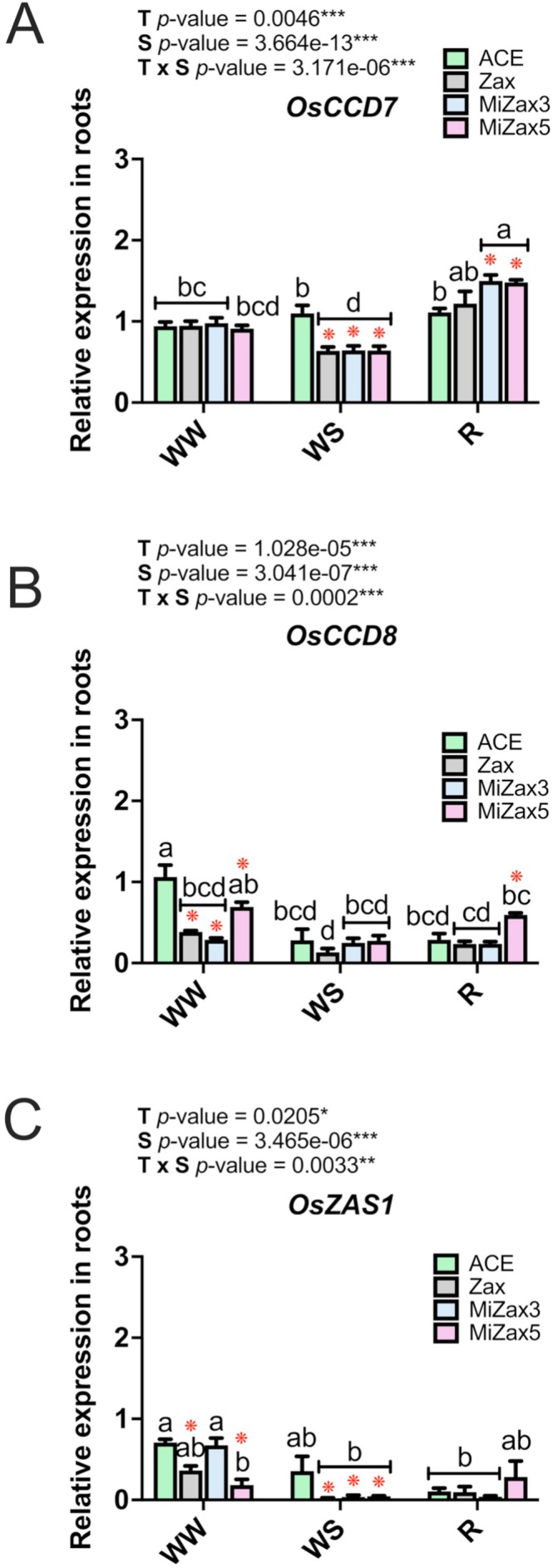
Relative expression levels of SL and zaxinone biosynthetic genes in the roots. Strigolactone (*OsCCD7* and *OsCCD8*) and zaxinone biosynthetic (*OsZAS1*) genes in roots of rice plants under well‐watered (WW) and water‐stressed (WS) conditions and at the last day of recovery (R). Treatments are reported in greyscale according to the color legend to the right of the graph (ACE, acetone control; Zax, zaxinone; MiZax3 and MiZax5, synthetic zaxinone mimics). All results are reported as mean ± SE. Statistically significant differences were determined using two‐way ANOVA, considering the interaction between treatment (T) and stress condition (S). Lowercase letters above the bars denote significant differences, assessed by the Tukey's HSD test (*p* < 0.05), and are reported only when the T × S interaction is significant. ns, not significant. Red asterisks indicate significant differences compared to control plants (ACE) within each stress condition (WW, WS, R), as assessed by the pairwise comparison *t*‐test (*p* < 0.05), as follows: *p ≤ 0.05, **p ≤ 0.01, ***p ≤ 0.00. Analysis of variance on the single variables is reported in Tables S9 and S10.

To assess the impact of the treatments on SLs and zaxinone biosynthesis, the relative expression levels of the SL biosynthetic genes *OsCCD7* (*LOC_Os11g12740*) and *OsCCD8* (*LOC_Os01g51690*) were evaluated in rice roots (Figure 5A,B; Table S9) and leaves (Figure S4; Table S10) under the different water conditions and treatments. *OsZAS1* (*LOC_Os09g15240*), which encodes the rice *Zaxinone Synthase 1* (Wang et al. 2019; Ablazov et al. 2023), was evaluated only in roots, based on previous experimental evidence showing its poor expression in leaf tissues (Wang et al. 2019). In roots, under WW conditions, *OsCCD7* expression showed no differences among the considered treatments (Figure 5A). Water stress induced a significant *OsCCD7* down‐regulation in plants treated with Zax and with both the MiZax molecules in comparison to ACE (Figure 5A). During recovery, *OsCCD7* expression levels increased in all treated plants, showing the highest expression values upon MiZax3 and MiZax5 treatments. In contrast, no statistically significant differences were detected in *OsCCD7* expression profiles in leaves of the same plants (Figure S4), except for WW MiZax3 samples, in which the gene underwent a significant downregulation with respect to WW ACE. Upon WW, *OsCCD8* transcript levels were higher in ACE plants with respect to all the other treatments and also in comparison to WS and R conditions. *OsCCD8* expression was poorly perturbed by either WS or R conditions, apart from plants exposed to MiZax5, for which an increased expression level was noticed under R conditions (Figure 5B). In leaves, no differences were evident among treatments or stress conditions. However, a significant effect was observed only for the interaction between these factors (T × S; Figure S4B). Looking at *OsZAS1* expression patterns in WW plants, the application of Zax and MiZax5 led to lower transcriptional rates with respect to treatment with ACE and MiZax3. Similar to *OsCCD7*, *OsZAS1* was strongly downregulated under WS conditions upon all treatments, while no differences occurred during recovery (Figure 5C). Overall, our results indicate that Zax and MiZax treatments differentially regulate the expression of SL biosynthetic transcripts in a gene‐ and water regime–dependent manner. Water stress downregulated *OsCCD7* and *OsZAS1* expression, whereas *OsCCD8* was unaffected by WS, but downregulated under WW. Notably, MiZax5 treatment enhanced the expression of both *OsCCD7* and *OsCCD8* during recovery.

## 4 Discussion

In comparison to other cereal species, rice is particularly sensitive to low soil moisture, probably as it has traditionally been cultivated under flooded irrigation conditions (Todaka et al. 2015; Hassan et al. 2023). Drought stress is a major constraint on rice productivity, causing substantial economic losses that are expected to become more severe with increasing climate variability (Turral 2011; Panda et al. 2021). In this study, we evaluated the response of potted rice plants to severe water deficit, following chemical priming with zaxinone and its mimics. These molecules have previously demonstrated promising growth‐promoting effects across various crops under both optimal growth conditions (Wang et al. 2020, 2022) and challenging environments (Wang, Jamil, et al. 2023; Wang, Zou, et al. 2023). Plant responses to drought are inherently complex, involving the coordinated action of diverse molecular, biochemical, and physiological mechanisms (Kumar et al. 2018). Here, the analysis of ecophysiological responses during the experimental trial indicated a strong decrease in *g*_*s*_ and *E* rates of WS plants, confirming the impact of the applied stress on the leaf transpiration dynamics. At the onset of stress (D_0_), Zax‐treated plants exhibited the highest stomatal conductance (*g*_*s*_), while MiZax5‐treated plants showed the greatest transpiration (*E*) rates. After 3 days of water deprivation (D_3_), plants treated with Zax, MiZax3, and MiZax5 had higher *g*_*s*_ values compared to the control (ACE). However, no significant differences were observed at the end of the stress period (D_6_), suggesting that these priming treatments might not provide long‐term protection against water stress, at least in terms of gas exchange maintenance. Reduced transpiration in stressed plants leads to decreased CO_2_ and nutrient uptake, limiting photosynthesis and impairing shoot growth (Ahluwalia et al. 2021). Chlorophyll content is closely linked to photosynthetic efficiency and organic matter accumulation. Our data on CCI correlated with *g*_*s*_ measurements, showing that stress timing (D) significantly affected chlorophyll levels, while treatment (T) had no impact. Specifically, the CCI in WS plants was halved compared to WW plants, with a full recovery observed at the end of the rehydration period (R). The decrease in CCI under water deficit is also in agreement with that previously observed by Adjah et al. (2025), considering diverse rice genotypes. Plant–water relations are quantitatively characterized by parameters such as leaf water potential (*Ψ*_stem_) and RWC, which serve as key indicators of plant hydration status (Farooq et al. 2009). No significant changes were observed in the soil water loss dynamics by calculations of RWCpot. By contrast, at the end of the WS period, MiZax5‐treated plants showed significantly higher values of *Ψ*_stem_ than ACE, suggesting that MiZax5 may improve the osmotic adjustment capacity compared to non‐treated plants.

To inspect how different treatments affect stress‐associated signals at the molecular level, changes in the expression of key genes involved in the regulation of defense responses to environmental stresses were considered. Among them, *OsLEA3*, a late embryogenesis abundant gene associated with osmotic stress and ABA regulation (Xiao et al. 2007; Olvera‐Carrillo et al. 2011; Lou et al. 2017), was evaluated in both leaves and roots, as *LEA* proteins are broadly expressed in both plant organs (Hong‐Bo et al. 2005). *OsLEA3* displayed tissue‐ and treatment‐specific expression patterns under WS, with a notable downregulation in leaves, whereas an opposite, though non‐significant pattern was seen in roots. This may reflect differential tissue responses or indicate that *OsLEA3*, as part of a multigene family, may not be the primary isoform involved in WS tolerance under our conditions. Interestingly, MiZax5‐treated WS plants showed significant downregulation of *OsLEA3* in roots, in contrast to the increased expression in MiZax3‐treated leaves. These contrasting patterns may suggest that the two treatments modulate stress perception differently, supported by the higher stem water potential observed in MiZax5‐treated plants under WS. We also examined *OsP5CS1*, a marker for proline accumulation and osmotic adjustment (Forlani et al. 2019; Shrestha et al. 2022). While MiZax5 downregulated *OsP5CS1* under WS, it significantly induced its expression under WW and R conditions, suggesting a priming effect that enhances the plant's capability to cope with water deficit, particularly by facilitating the recovery dynamics. This hypothesis is further supported by the expression of ABA biosynthetic genes: *OsNCED3*, *OsNCED4*, and *OsNCED5* (Changan et al. 2023; Huang et al. 2018, 2019; Xiang et al. 2024). MiZax5 treatment upregulated *OsNCED3* and *OsNCED4* under WW in leaves, while under WS, it enhanced *OsNCED4*, *OsNCED5*, and *OsABAox1* expression in roots, indicating organ‐specific ABA pathway modulation. Consistent with our findings, *OsNCED4* has been implicated in enhancing tolerance to multiple abiotic stresses in rice, as CRISPR/Cas9‐mediated disruption of this gene resulted in heightened sensitivity to both salt (NaCl) and cold stress (Xiang et al. 2024). Despite *OsNCED3* being typically stress‐inducible (Ye et al. 2012), it was downregulated under WS in our study, likely reflecting different stress timing or intensity. Conversely, MiZax3‐treated plants exhibited in WW conditions an increased expression of *OsNCED5* in leaves and enhanced the level of *OsNCED4* and *OsNCED5* in roots. Regarding zaxinone application, no significant differences in the expression of ABA biosynthetic genes were observed in leaves under WW. However, a significant downregulation of *NCED3* and *NCED4* was recorded in roots, in contrast with the findings by Wang et al. (2019) showing no changes in the transcriptional profiles of those genes in rice seedlings under WW conditions. Such discrepancies could be attributed to differences in plant age, timing of compound application, and growth conditions. During recovery, all treatments downregulated *OsNCED3*, *OsNCED4*, and *OsNCED5* in both tissues, pointing to a common role in restoring hormonal balance. Additionally, *OsABAox1*, involved in ABA degradation, was significantly induced by WS in all treated leaves, suggesting enhanced catabolism. Its strong upregulation in non‐treated plants during recovery aligns with its known rehydration‐induced role (Ye et al. 2012), while the attenuated expression in treated plants indicates modified ABA dynamics under MiZax application. Together, these data reveal distinct and compound‐specific regulations of ABA metabolism and stress‐responsive gene expression, supporting a role for MiZax5 in stress priming and post‐stress recovery, and highlighting the complexity of ABA signaling modulation in rice under fluctuating water availability.

The increase in the ABA level under environmental stress conditions can have an impact on the biosynthesis of ethylene, another phytohormone involved in stress response (Wilkinson and Davies 2010; Müller 2021). Indeed, the expression of an *OsACO* gene, coding for *1‐aminocyclopropane‐1‐carboxylic acid oxidase* involved in the last stage of ethylene biosynthesis, has been considered due to its potential role in modulating the antagonistic interaction between ABA and ethylene during abiotic stress responses (Müller 2021). *OsACO* expression resulted to be higher in all stressed plants treated with the three compounds compared to non‐treated plants, whereas the differences were statistically significant only for zaxinone and MiZax3. Notably, the role of ethylene in stomatal closure remains controversial and appears to be species‐dependent. For example, evidence in *Arabidopsis thaliana* indicates that ethylene can promote stomatal closure by triggering hydrogen peroxide (H_2_O_2_) production via an ABA‐independent pathway (Desikan et al. 2006; Daszkowska‐Golec and Szarejko 2013). A different trend was observed in the roots, where zaxinone and MiZax5 induced an accumulation of ABA content compared to ACE under WS conditions. Under WW conditions, zaxinone content in roots decreased following treatment, likely due to a negative feedback mechanism on *OsZAS1* expression triggered by exogenous zaxinone or its mimics. Moreover, considering WS conditions, our findings suggested a novel putative role for zaxinone in rice water stress regulation. Indeed, in the root of non‐treated (ACE) plants, *OsZAS1* expression levels and zaxinone content decreased, in contrast to ABA content. These results pointed out a potential negative crosstalk between ABA and zaxinone under WS conditions, which may occur either directly or be mediated by SLs. SL‐deficient mutants in Arabidopsis, *Lotus*, and tomato exhibit increased drought sensitivity, regardless of shoot ABA levels (Ha et al. 2014; Liu et al. 2015; Visentin et al. 2016; Cardinale et al. 2018). In barley, impaired ABA signaling in the SL‐insensitive *hvd14.d* mutant supports a role for SL–ABA interplay in drought responses (Marzec et al. 2020). Conversely, in *Lotus* and tomato, exogenous SL treatment suppressed ABA accumulation under drought, highlighting species‐ or context‐specific SL–ABA interactions (Liu et al. 2015; Visentin et al. 2016).

Wang et al. (2019, 2020) demonstrated that zaxinone and its mimics negatively regulate SLs biosynthesis. The key genes (*OsCCD7* and *OsCCD8*) involved in SLs biosynthesis were tested under zaxinone and MiZax treatments in WS, WW and R conditions. In our set‐up, *CCD7* was not differentially regulated in ACE‐treated roots across all water conditions, while *CCD8* transcript levels decreased in WS and R conditions compared to WW. On the other hand, water availability did not significantly affect the expression of both genes in the shoots of ACE‐treated plants, unlike in tomato, where drought upregulated their expression in shoots (Visentin et al. 2016). Regarding the effect of exogenous zaxinone and its mimics on their gene expression, we observed in the roots that *CCD7* was downregulated under WS conditions, while *CCD8* expression was reduced under WW conditions, confirming the negative impact of these compounds on SLs biosynthesis. By the end of the recovery, however, MiZax5 treatment induced both *CCD7* and *CCD8* transcript levels. Notably, during the recovery phase after drought stress, the tomato *CCD7* mutant exhibited an incomplete restoration of stomatal conductance, although the water potential was fully recovered (Visentin et al. 2020, 2025). These findings suggest that MiZax5 may enhance stress memory and prime rice plants for improved drought resilience by upregulating SL biosynthetic gene expression after the plants are exposed to water stress.

**FIGURE 6:**
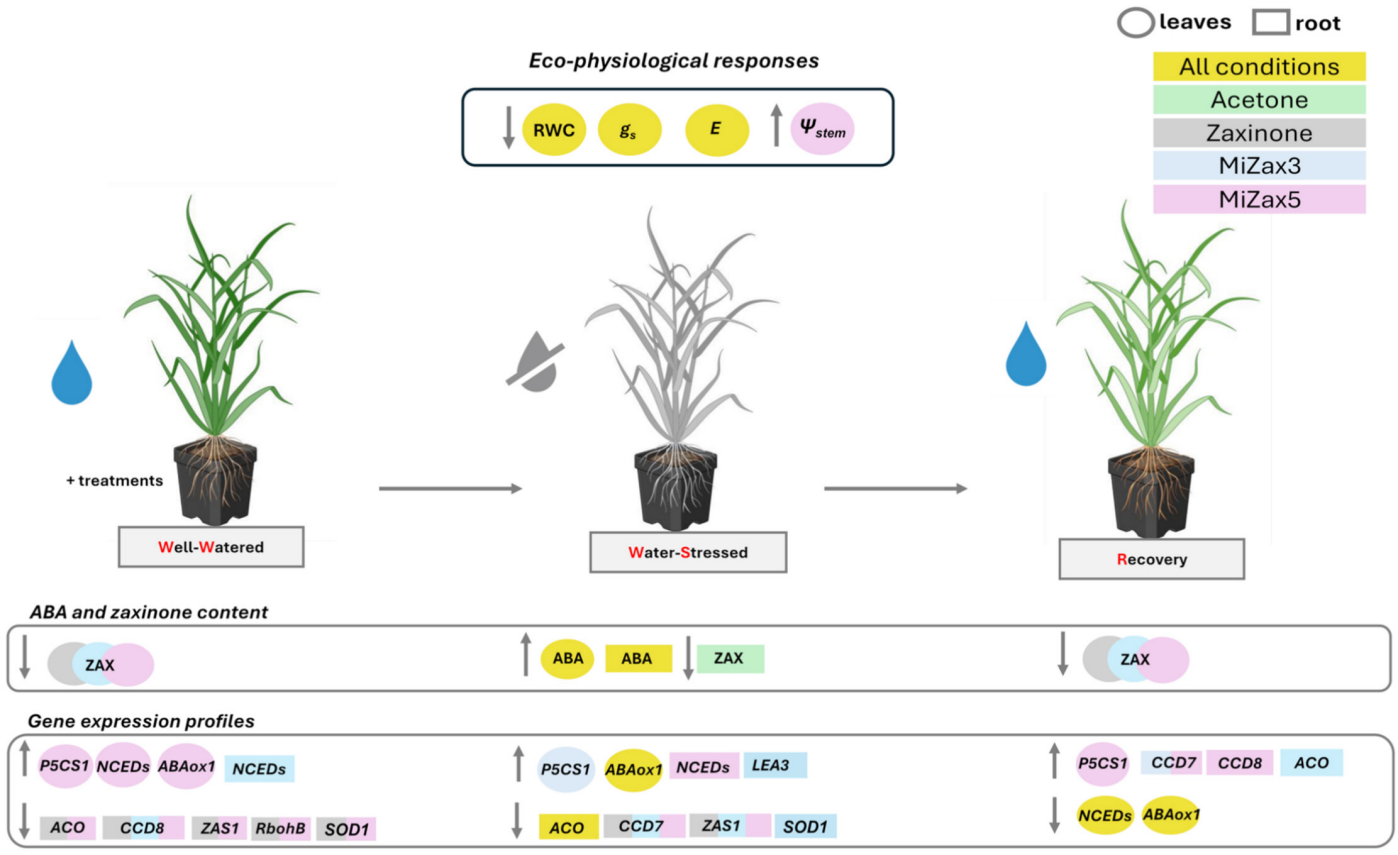
Graphical summary illustrating the experimental setup and the main results obtained under three watering conditions in control and in treated plants. Data were evaluated in both leaves (circle) and roots (rectangle). An upward arrow indicates an increase, while a downward arrow indicates a decrease. Data highlighted in yellow showed the same trend under all conditions (acetone, zaxinone, MiZax3, MiZax5), in green significantly modulated by acetone, in grey by zaxinone, in blu by MiZax3 and in purple by MiZax5. ABA, Abscisic acid; *ABAox1*, *ABA 8‐hydroxylase*; *ACO*, *1‐aminocyclopropane‐1‐carboxylate oxidase*; *CCD7/CCD8*, *Carotenoid cleavage dioxygenase 7/8*; E, Leaf transpiration; *g*_*s*_, Stomatal conductance; *LEA3*, *Late Embryogenesis Abundant*; *NCEDs*, *9‐cis‐epoxycarotenoid dioxygenase gene family*; *P5CS1*, *Δ* ^*1*^ *‐pyrroline‐5‐carboxylate synthetase 1*; *RbohB*, *Respiratory Burst Oxidase Homolog B*; RWC, Percentage of soil relative water content; *SOD*, *Superoxide Dismutase; ZAS*, *Zaxinone synthase*; ZAX, Zaxinone; *Ψ*_stem_, Stem water potential.

In conclusion, although zaxinone and its mimics similarly influence rice architecture and suppress SL biosynthesis under optimal conditions, their effects under water stress differ by organ and water regime (Figure 6). Although all tested compounds increased stomatal conductance relative to untreated controls, MiZax5 consistently exhibited the most pronounced positive effects, enhancing drought tolerance by elevating stem water potential and regulating stress‐responsive gene expression. Furthermore, MiZax5 may support drought stress memory through the reactivation of SL biosynthetic pathways. Altogether, these results underscore the potential of zaxinone‐derived compounds as sustainable, eco‐friendly biostimulants that enhance crop performance and prime plants for improved drought resilience, advancing strategies for climate‐resilient agriculture.

## Author Contributions

T.M., L.G., C.V., and C.P. performed the biometric and eco‐physiological analyses. G.‐T.E.C., J.Y.W., and T.A. provided the molecules and conducted metabolite extraction and quantification. T.M. and L.G. carried out the quantitative gene expression analysis. V.F., R.B., S.A.‐B., and L.L. designed and planned the research. V.F. and R.B. wrote the manuscript. V.F., R.B., J.Y.W., L.L., and S.A.‐B. revised and edited the manuscript. All authors approved the final version.

## Funding

This work was supported by the European Union ‐ NextGenerationEU (PRIN prot. 2022CWZNZC to VF), by the Competitive Research Grant CRG 2020 given to SA‐B and LL from King Abdullah University of Science and Technology and by the project CN_00000033 funded under the National Recovery and Resilience Plan (NRRP), Mission 4 Component 2 Investment 1.4 ‐ Call for tender No. 3138 of 16 December 2021, rectified by Decree n. 3175 of 18 December 2021 of the Italian Ministry of University and Research funded by the European Union ‐ NextGenerationEU. This study was also carried out within the Agritech National Research Center and received funding from the European Union Next ‐ Generation EU (Piano Nazionale Di Ripresa E Resilienza (PNRR) – Missione 4 Componente 2, Investimento 1.4 – D.D. 1032 17/06/2022, CN00000022). The manuscript reflects only the authors' views and opinions, and neither the European Union nor the European Commission can be considered responsible for them.

## Conflicts of Interest

The authors declare no conflicts of interest.

## Supporting information


**Figure S1:** Effects of treatment with Zax and synthesized mimics (MiZax3 and MiZax5) on the physiological performance of potted rice plants ( *Oryza sativa* subsp. *japonica* cv Nipponbare).
**Figure S2:** Effects of treatments with zaxinone (Zax) and synthesized mimics (MiZax3 and MiZax5) on the biometric parameters.
**Figure S3:** Relative expression of oxidative stress‐related genes in rice leaves and roots.
**Figure S4:** Relative expression levels of genes involved in strigolactone biosynthesis (*OsCCD7*, *OsCCD8*) in leaves of potted rice plants on the last day of stress (October 2, 2023), under well‐watered (WW) and water‐stressed (WS) conditions and at the last day of recovery (R; October 10, 2023).

**Table S1:** Primer sequences used in this study.
**Table S2:** Effects of treatments with Zax and synthesized mimics (MiZax3 and MiZax5) on the physiological performance of potted rice plants ( *Oryza sativa* subsp. Japonica cv. Nipponbare). Dynamic changes in stomatal conductance (*gs*, mol H_2_O m^−2^ s^−1^) and leaf transpiration (*E*, mmol H_2_O m^−2^ s^−1^) monitored throughout the entire duration of the water stress period and subsequent recovery (D_0_–D_14_). All results are presented as means ± SE. Statistically significant differences were determined using one‐way ANOVA. Different lowercase letters indicate significant differences among groups and are reported only when the treatment (T) is significant, as assessed by Tukey HSD test (*p* < 0.05). ns, not significant; *p ≤ 0.05, **p ≤ 0.01, ***p ≤ 0.001. ACE, control; MiZax3 and MiZax5, synthesized mimic treatments; WS, water stress; WW, well‐watered; Zax, zaxinone treatment.
**Table S3:** Effects of treatments with Zax and synthesized mimics (MiZax3 and MiZax5) on the physiological performance of potted rice plants ( *Oryza sativa* subsp. Japonica cv. Nipponbare) during the water stress (WS) period and subsequent rehydration (R). Stomatal conductance (*g*_*s*_ mol H_2_O m^−2^ s^−1^) and leaf transpiration (*E*, mmol H_2_O m^−2^ s^−1^) rates measured at the beginning (D_0_), at the end of the stress period (D_6_), and on the final day of recovery (D_14_). All results are presented as means ± SE. Statistically significant differences were determined using two‐way ANOVA, considering the interaction between treatment (T) and days (D). Different lowercase letters indicate significant differences among groups and are reported only when the T × D interaction is significant, as assessed by Tukey HSD test (*p* < 0.05). ns, not significant; *p ≤ 0.05, **p ≤ 0.01, ***p ≤ 0.001. ACE, control; MiZax3 and MiZax5, synthesized mimic treatments; WW, well‐watered; WS, water stress; Zax, zaxinone treatment.
**Table S4:** Effects of treatments with Zax and synthesized mimics (MiZax3 and MiZax5) on the physiological performance of potted rice plants ( *Oryza sativa* subsp. Japonica cv. Nipponbare). Stomatal conductance (*gs* mol H_2_O m^−2^ s^−1^) and leaf transpiration (*E*, mmol H_2_O m^−2^ s^−1^) rates measured at the last day of stress (D_6_), after 2 days of rehydration (D_8_) and at the end of the recovery period (D_14_). All results are presented as means ± SE. Statistically significant differences were determined using two‐way ANOVA, considering the interaction between treatment (T) and stress (S). Different lowercase letters indicate significant differences among groups and are reported only when the T × S interaction is significant, as assessed by Tukey HSD test (*p* < 0.05). ns, not significant; *p ≤ 0.05, **p ≤ 0.01, ***p ≤ 0.001. ACE, control; MiZax3 and MiZax5, synthesized mimic treatments; R, recovery; WW, well‐watered; WS, water stress; Zax, zaxinone treatment.
**Table S4:** Effects of treatments with Zax and synthesized mimics (MiZax3 and MiZax5) on the physiological performance of potted rice plants ( *Oryza sativa* subsp. Japonica cv. Nipponbare). Stem water potential (*Ψ*_stem_) measured at the end (D_6_; October 2, 2023) of the water stress (WS). All results are reported as mean ± SE, for WW conditions only biological replicates are reported. Asterisk denotes cases where a treatment significantly differed from the control (ACE), as determined by a pairwise comparison *t*‐test (*p* < 0.05). ns, not significant; *p ≤ 0.05, **p ≤ 0.01, ***p ≤ 0.001. ACE, control; MiZax3 and MiZax5, synthesized mimic treatments; WW, well‐watered; WS, water stress; R, recovery; Zax, zaxinone treatment.
**Table S6:** Effects of treatments with Zax and synthesized mimics (MiZax3 and MiZax5) on the biometric parameters, shoot dry weight (g) and root dry weight (g) of potted rice plants ( *Oryza sativa* subsp. Japonica cv. Nipponbare) at the last day of stress (October 2, 2023), under well‐watered (WW) and water‐stressed (WS) conditions and at the last day of recovery (R; October 10, 2023). All results are reported as mean ± SE. Statistically significant differences were determined using two‐way ANOVA, considering the interaction between treatment (T) and stress condition (S). Lowercase letters above the bars denote significant differences, assessed by Tukey HSD test (*p* < 0.05) and are reported only when the T × S interaction is significant. Asterisks indicate significant differences compared to control plants (ACE) within each stress condition (WW, WS, R), as assessed by the pairwise comparison *t*‐test (*p* < 0.05). ns, not significant; *p ≤ 0.05, **p ≤ 0.01, ***p ≤ 0.00. ACE, control; R, recovery; WW, well‐watered; WS, water stress; MiZax3 and MiZax5, synthesized mimic treatments; Zax, zaxinone treatment.
**Table S7:** Effect of treatments with Zax and synthesized mimics (MiZax3 and MiZax5) on Chlorophyll Content Index (CCI, °SPAD) of potted rice plants ( *Oryza sativa* subsp. Japonica cv. Nipponbare) at the beginning of the stress (D_0_; September 26, 2023), the end of the stress (D_6_; October 2, 2023), and the final day of the recovery (D_14_; October 10, 2023). All results are reported as mean ± SE. Statistically significant differences were determined using two‐way ANOVA, considering the interaction between treatment (T) and days (D). Lowercase letters above the bars denote significant differences, assessed by Tukey HSD test (*p* < 0.05) and are reported only when the T × D interaction is significant. Asterisks indicate significant differences compared to control plants (ACE) within each stress condition (WW, WS, R), as assessed by the pairwise comparison t‐test (*p* < 0.05). ns, not significant; *p ≤ 0.05, **p ≤ 0.01, ***p ≤ 0.00. ACE, control; MiZax3 and MiZax5, synthesized mimic treatments; R, recovery; WW, well‐watered; WS, water stress; Zax, zaxinone treatment.
**Table S8:** Phytohormone content in leaves and roots of potted rice plants ( *Oryza sativa* subsp. Japonica cv. Nipponbare) sampled at the last day of stress (October 2, 2023), under well‐watered (WW) and water‐stressed (WS) conditions and at the last day of recovery (R; October 10, 2023). Abscisic acid (ABA) concentrations in leaves and roots and zaxinone (Zax) concentrations in leaves and roots. Data are expressed as pg/mg DW. All results are reported as mean ± SE. Statistically significant differences were determined using two‐way ANOVA, considering the interaction between treatment (T) and stress condition (S). Lowercase letters above the bars denote significant differences, assessed by Tukey HSD test (*p* < 0.05) and are reported only when the T × S interaction is significant. Asterisks indicate significant differences compared to control plants (ACE) within each stress condition (WW, WS, R), as assessed by the pairwise comparison t‐test (*p* < 0.05). ns, not significant; *p ≤ 0.05, **p ≤ 0.01, ***p ≤ 0.00. ACE, control; MiZax3 and MiZax5, synthesized mimic treatments; R, recovery; WW, well‐watered; WS, water stress; Zax, zaxinone treatment.
**Table S9:** Relative expression levels of selected genes tested in leaves of potted rice plants ( *Oryza sativa* subsp. Japonica cv. Nipponbare) at the last day of stress (October 2, 2023), under well‐watered (WW) and water‐stressed (WS) conditions and at the last day of recovery (R; October 10, 2023). All results are reported as mean ± SE. Statistically significant differences were determined using two‐way ANOVA, considering the interaction between treatment (T) and stress condition (S). Lowercase letters above the bars denote significant differences, assessed by Tukey HSD test (*p* < 0.05) and are reported only when the T × S interaction is significant. Asterisks indicate significant differences compared to control plants (ACE) within each stress condition (WW, WS, R), as assessed by the pairwise comparison *t*‐test (*p* < 0.05). ns, not significant; *p ≤ 0.05, **p ≤ 0.01, ***p ≤ 0.00. ACE, control; MiZax3 and MiZax5, synthesized mimic treatments; R, recovery; WS, water stress; WW, well‐watered; Zax, zaxinone treatment.
**Table S10:** Relative expression levels of selected genes tested in roots of potted rice plants ( *Oryza sativa* subsp. Japonica cv. Nipponbare) at the last day of stress (October 2, 2023), under well‐watered (WW) and water‐stressed (WS) conditions and at the last day of recovery (R; October 10, 2023). All results are reported as mean ± SE. Statistically significant differences were determined using two‐way ANOVA, considering the interaction between treatment (T) and stress condition (S). Lowercase letters above the bars denote significant differences, assessed by Tukey HSD test (*p* < 0.05) and are reported only when the T × S interaction is significant. Asterisks indicate significant differences compared to control plants (ACE) within each stress condition (WW, WS, R), as assessed by the pairwise comparison *t*‐test (*p* < 0.05). ns, not significant; *p ≤ 0.05, **p ≤ 0.01, ***p ≤ 0.00. ACE, control; R, recovery; MiZax3 and MiZax5, synthesized mimic treatments; WS, water stress; WW, well‐watered; Zax, zaxinone treatment.

## Data Availability

The data are available in the article.
