# Supplementary material for: Boosting Drought Resilience in Rice: The Priming Effects of Zaxinone and Its Mimics
Source: Physiol Plant. 2025 Nov 30;177(6):e70667. doi: 10.1111/ppl.70667 (PMC12666242; doi:10.1111/ppl.70667)
Supplement: Supplementary file 1 — Figure S1: Effects of treatment with Zax and synthesized mimics (MiZax3 and MiZax5) on the physiological performance of potted rice plants ( Oryza sativa subsp. japonica cv Nipponbare). Figure S2: Effects of treatments with zaxinone (Zax) and synthesized mimics (MiZax3 and MiZax5) on the biometric parameters. Figure S3: Relative expression of oxidative stress‐related genes in rice leaves and roots. Figure S4: Relative expression levels of genes involved in strigolactone biosynthesis (OsCCD7, OsCCD8) in leaves of potted rice plants on the last day of stress (October 2, 2023), under well‐watered (WW) and water‐stressed (WS) conditions and at the last day of recovery (R; October 10, 2023). [file PPL-177-e70667-s001.pdf]

Supplementary figures

Boosting drought resilience in rice: the priming effects of zaxinone and its mimics

Teresa Mazzarella [1‡], Luca Giovannini [2‡], Guan-Ting Erica Chen [3], Cristina Votta [1], ChiaraPagliarani [2], Jian You Wang [3§], Tadao Asami [4], Luisa Lanfranco [1], Salim Al-Babili [3,5], Raffaella Balestrini[6*], Valentina Fiorilli [1*].

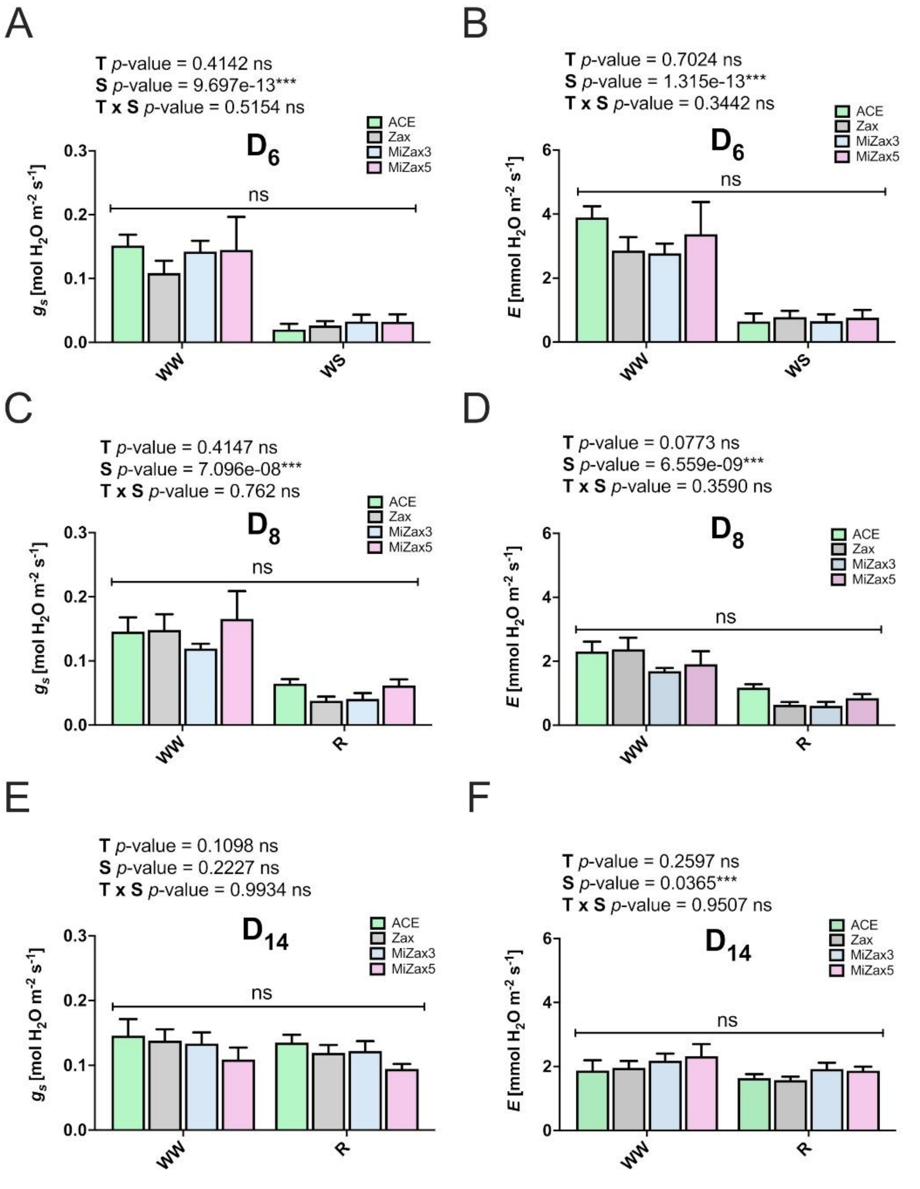

Figure S1: Effects of treatment with Zax and synthesized mimics (MiZax3 and MiZax5) on the physiological performance of potted rice plants (*Oryza sativa* subsp *japonica* cv Nipponbare).

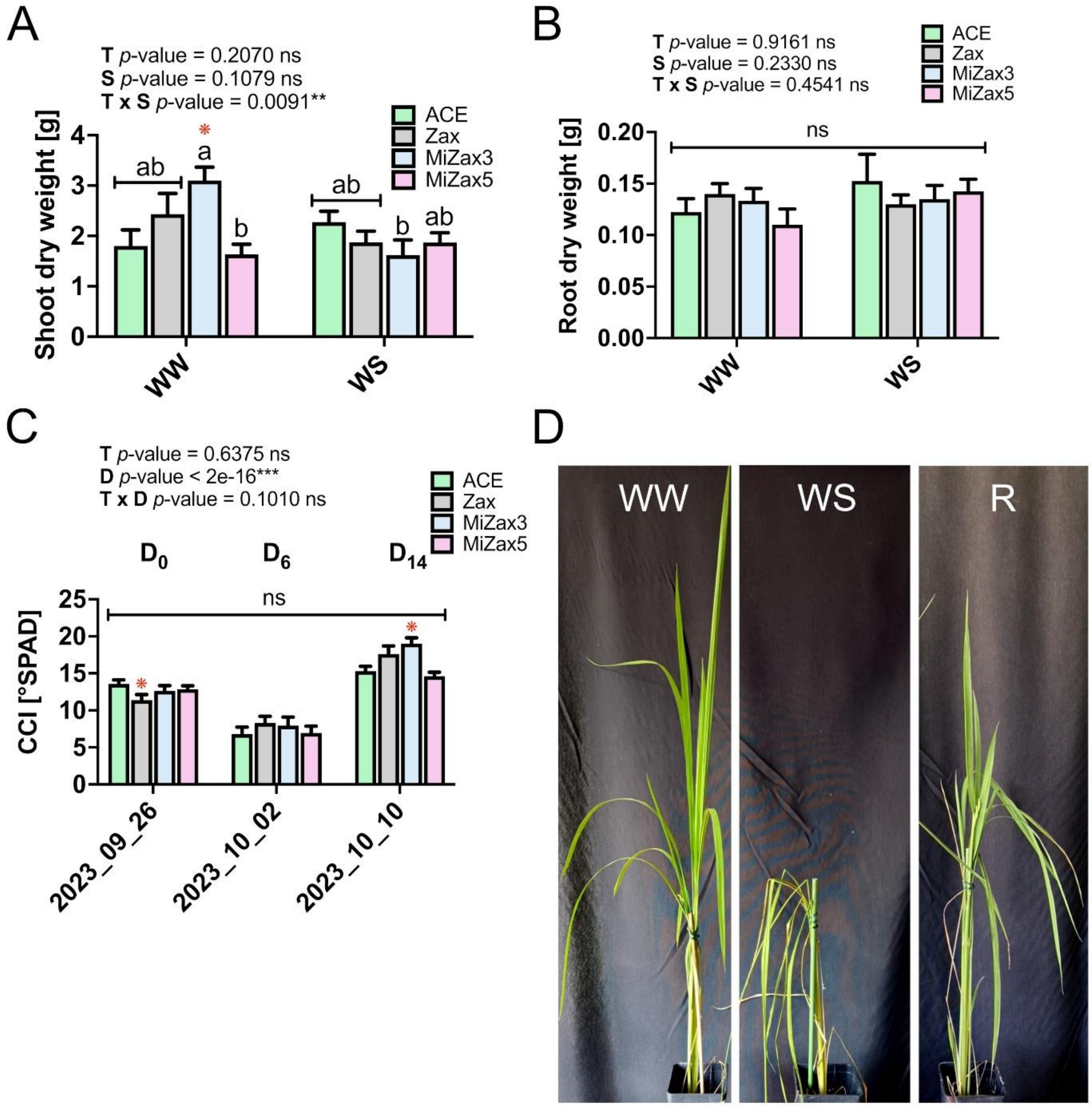

Figure S2. Effects of treatments with zaxinone (Zax) and synthesized mimics (MiZax3 and MiZax5) on the biometric parameters.

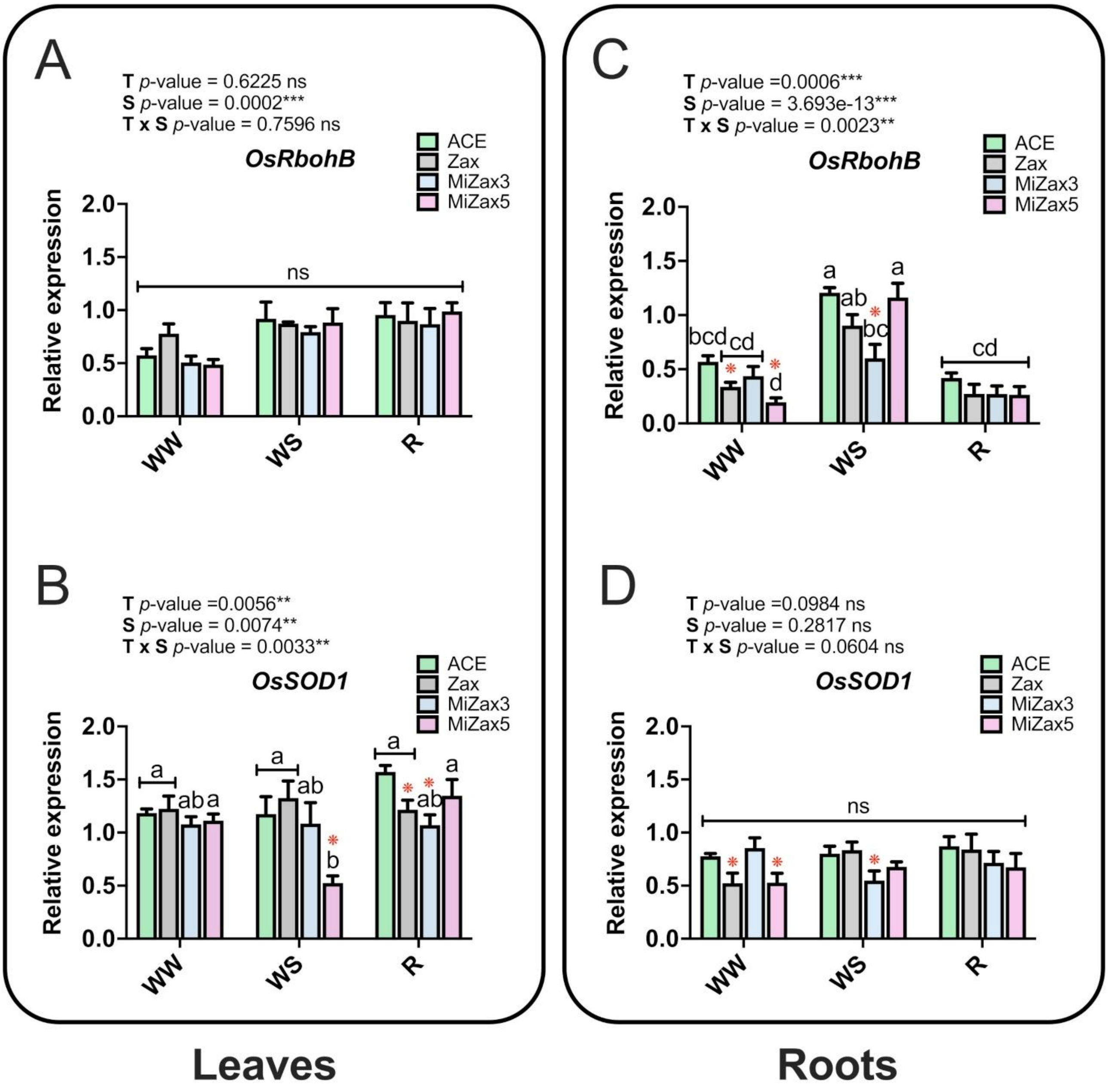

Figure S3. Relative expression of oxidative stress-related genes in rice leaves and roots.

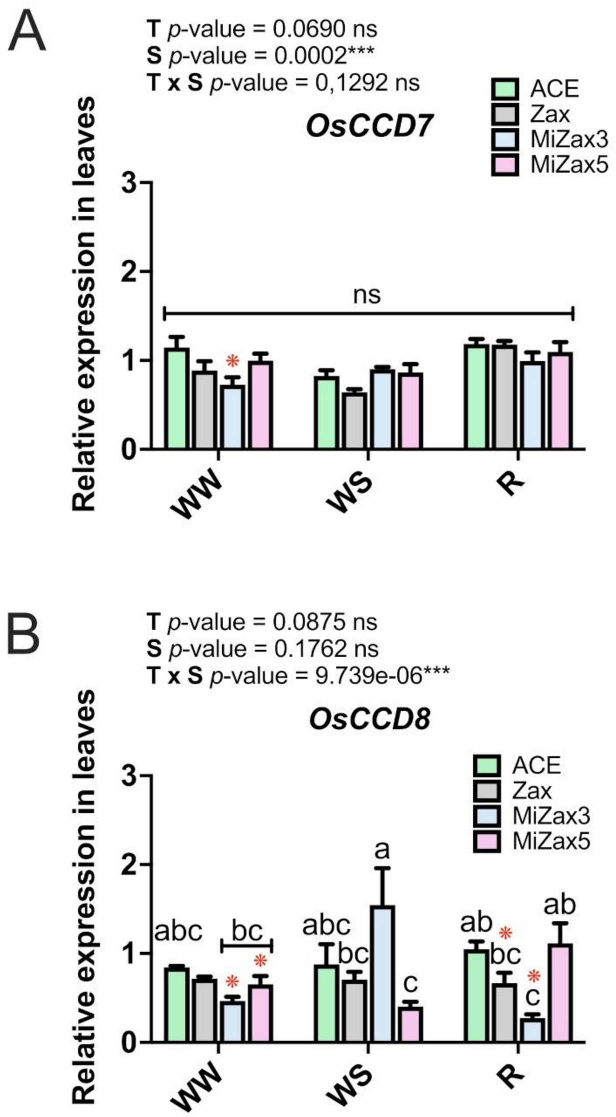

Figure S4. Relative expression levels of genes involved in strigolactone biosynthesis (*OsCCD7*, *OsCCD8*) in leaves of potted rice plants on the last day of stress (October 02, 2023), under well-watered (WW) and water-stressed (WS) conditions and at the last day of recovery (R; October 10, 2023).
